# User guide for mapping-by-sequencing in *Arabidopsis*

**DOI:** 10.1186/gb-2013-14-6-r61

**Published:** 2013-06-17

**Authors:** Geo Velikkakam James, Vipul Patel, Karl JV Nordström, Jonas R Klasen, Patrice A Salomé, Detlef Weigel, Korbinian Schneeberger

**Affiliations:** 1 Max Planck Institute for Plant Breeding Research, Department for Plant Developmental Biology, 50829 Cologne, Germany; 2 Department of Biological Sciences, Institute of Genetics/Epigenetics, University of Saarland, Campus Saarbrücken, 66123 Saarbrücken, Germany; 3 Max Planck Institute for Developmental Biology, Department of Molecular Biology, 72076 Tübingen, Germany

**Keywords:** Mapping-by-sequencing, SHOREmapping, genetic mapping, whole-genome sequencing, *Arabidopsis thaliana*

## Abstract

Mapping-by-sequencing combines genetic mapping with whole-genome sequencing in order to accelerate mutant identification. However, application of mapping-by-sequencing requires decisions on various practical settings on the experimental design that are not intuitively answered. Following an experimentally determined recombination landscape of *Arabidopsis *and next generation sequencing-specific biases, we simulated more than 400,000 mapping-by-sequencing experiments. This allowed us to evaluate a broad range of different types of experiments and to develop general rules for mapping-by-sequencing in *Arabidopsis*. Most importantly, this informs about the properties of different crossing scenarios, the number of recombinants and sequencing depth needed for successful mapping experiments.

## Background

Forward genetic screens remain one of the major genetic tools to uncover gene function in plants as well as in other organisms. However, genetic mapping, the process that links a phenotype to its causal mutation, is tedious and time-consuming.

Recently, the combination of bulk segregant analysis and whole-genome resequencing has proven to radically speed up this process [1]. This speed-up was gained by whole-genome sequencing of bulked recombinants and a subsequent analysis for local skews in the parental allele frequencies, which are introduced through phenotypic selection for mutant phenotypes. This directs the analysis to an approximate mapping interval that can be screened for underlying mutations using the exact same sequencing data. Several analysis pipelines have been introduced [1-9] and were already applied to various model species, including plants, yeast, nematodes, mammals, and invertebrates [10-12].

Different types of crossing schemes for mapping-by-sequencing have been suggested. The very first mapping-by-sequencing experiments were performed on pooled genomes of mutant recombinants that were generated by crossing the mutants to diverged strains followed by one round of selfing [1,11]. Recently, several groups suggested using backcrossed instead of outcrossed individuals as mapping populations, as mutagen-induced changes segregate like natural polymorphisms. Even though there is no prior knowledge about their distribution or location, mutagen-induced changes can be identified within whole-genome sequencing data and subsequently used for mapping [5,6,13]. Similarly, direct sequencing of an individual mutant recombinant, as suggested for *Caenorhabditis elegans *and later for *Arabidopsis thaliana *(Arabidopsis), will allow for a rough mapping of the causal mutation [14,15]. Although multiple rounds of backcrossing are usually not sufficient to considerably minimize the size of linked regions around causal mutations, this strategy has the advantage to characterize the complete genome of a mutant recombinant. Alternatively, direct sequencing of two or more independently generated alleles of the same mutant followed by a subsequent search for genes that carry mutations in all mutant alleles is powerful enough to unambiguously identify the causal mutation [16].

Irrespective of the actual strategy, application of mapping-by-sequencing involves decisions on the experimental makeup, for instance the size of the mapping population, as well as the amount of next generation sequencing data. Since both are directly related to time and financial effort, it is important to optimize the setup of mapping-by-sequencing experiments. The lack of general guidelines describing an optimal design might lead to conservative decisions that prime an unnecessarily high number of individuals and sequencing coverage.

Within this study, we establish a guideline for mapping-by-sequencing for *Arabidopsis*. Following an experimentally established recombination landscape [17], we simulated next generation sequencing of >400,000 mapping-by-sequencing experiments to analyze the differences in the design of mapping populations in relation to the number of candidate mutations identified in the course of such an experiment. Furthermore, we evaluated the impact of technical aspects, such as read length and read pairing, on mapping-by-sequencing.

Even though our simulations were focused on *Arabidopsis*, our simulation pipeline, called Pop-seq simulator, is generic and can be applied to other species as well as other mapping or sequencing strategies. In the last section, we describe the extension of our analysis on the experimental design of mapping-by-sequencing to two crop model species, rice and barley, in which next generation sequencing-based mapping becomes tangible reality.

## Results and Discussion

### *In-silico *mapping-by-sequencing experiments

Assessing different types of mapping-by-sequencing experiments requires establishment and sequencing of thousands of mapping populations, which is practically not feasible in plants. In contrast, *in-silico *simulations do allow for the generation of many experiments, with the potential caveat that they rely on prior assumptions. In particular, genuine simulations of mapping-by-sequencing experiments require realistic assumptions about mutation load, next generation sequencing, and meiotic recombination.

The most commonly used mutagen for *Arabidopsis *is ethyl-methanesulfonate (EMS), a chemical mutagen that predominantly introduces C to T and G to A changes. There are various reports about the frequency of EMS-induced mutations, including one change in 112 to one change in 171 kb [15,18], indicating a dosage dependency of the mutation rate, which suggests that the actual frequency range is likely to be much wider. In order to explore the effects of different mutation rates, we simulated low (700 changes) and high (1,400 changes) rates of mutations that were randomly introduced into the genome.

Similarly, realistic simulations of next generation sequencing rely on correct assumptions about the number of short read alignments per reference position (from here on referred to as coverage) and sequencing errors. As we were only interested in coverage at marker loci, we simulated whole-genome sequencing by randomizing the number of read alignments at each marker. The absolute number of alignments per marker followed a coverage distribution assessed on real resequencing experiments using Illumina sequencing. Deriving the coverage distribution from real sequencing experiments has the advantage that it considers all factors that contribute to the variation in sequence coverage. Perhaps most prominently, several different groups have demonstrated that local GC content is correlated with sequence coverage (for example, [19,20]), which is consequently also represented in our coverage landscape. Moreover, within a recent study we rigorously assessed the sequencing error rate of sequence reads aligned to marker positions [7], where the actual per base sequencing error rate was between 0.09% and 0.21% after quality filtering. In order to avoid overly optimistic simulation we assumed a sequencing error rate of 0.3% in our simulations. Based on these assumptions each of the simulated read alignments was then assigned to a parental allele, following a multinomial distribution based on local allele frequencies within the bulked segregants and Illumina sequencing-specific error rate [21] (Materials and methods).

Most important, however, might be realistic simulations of recombinant genomes that greatly rely on frequency and location of recombination. Thus, we based our simulations on experimentally determined recombination frequencies derived from a F_2 _population established by crossing two diverged *Arabidopsis *accessions [17]. These data reveals the number of recombination in single crosses as well as their distribution over the physical range of the chromosomes. We used the frequency of recombination events along the chromosomes as a probability function after which recombination location and frequency were simulated (Materials and methods).

**Figure 1:**
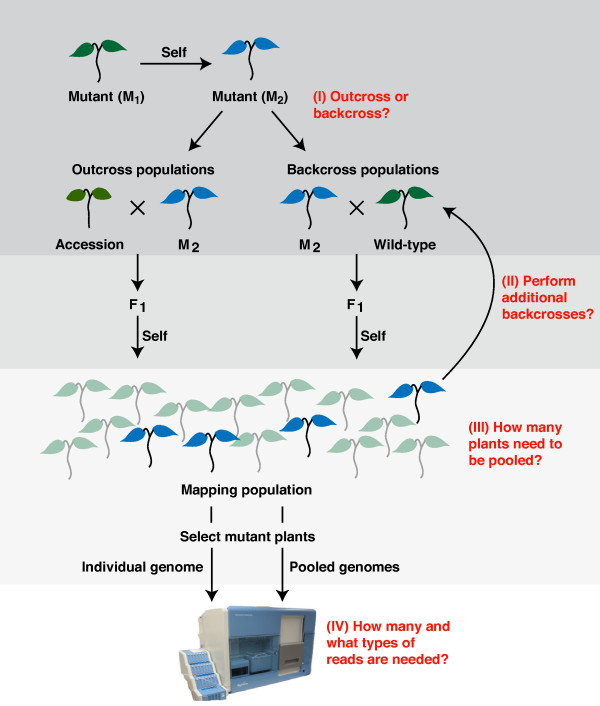
**Overview of different strategies for mapping-by-sequencing**. Establishing mutant pools or individual mutant recombinant genomes requires decisions on different aspects of mapping-by-sequencing. Common questions are shown in red. (I) Mutants can be crossed to diverged accessions or backcrossed to the wild-type. (II) The number of backcrosses and number of plants used as parents contribute to the outcome of mapping-by-sequencing. (III) The number of mutant plants sampled from mapping population greatly impacts on the mapping results. (IV) Finally, the sequencing coverage as well as type of sequencing (single-end or paired-end) affects the outcome of mapping-by-sequencing.

This method for *in-silico *simulation of recombination breakpoint events can be applied to any type of crossing regime. In this study, we focused on three different types of mapping-by-sequencing scenarios (Figure 1). First, we simulated F_2 _mapping populations generated by crossing a mutant plant to a non-mutagenized accession with a diverged background followed by selfing of the F_1 _hybrid (as performed by [1,3,11]). We refer to these classical mapping populations as 'outcross populations'. In outcross populations, natural sequence variations along with mutagen-induced changes serve as genetic markers. A second type of population was simulated by backcrossing the mutant plant to the non-mutagenized progenitor, followed by selfing of the hybrid (as performed by [5,6]). We refer to these mapping populations as 'backcross populations', in which only mutagen-induced changes serve as markers.

In contrast to the previous two methods, which make use of recombination, the third type of simulation constitutes direct sequencing of individual mutant genomes selected from the backcross populations (as performed by [14,15]).

In the next sections, we explore the consequences of different crossing schemes and the effect of pool size and coverage on the extent of the resulting mapping interval and on the number of candidate mutations (CAMs).

### Mapping-by-sequencing with outcross populations

Mapping-by-sequencing with outcross populations is based on mutant allele frequencies assessed at large-scale marker sets leading to the identification of mapping intervals. Such regions can then be screened for novel mutagen-induced changes using the same whole-genome sequencing data (see (I) in Figure 1). Usually a rough identification of linked regions suffices, as even in larger regions sequencing data can easily be screened for CAMs. In order to evaluate this process we used the mapping-by-sequencing analysis pipeline SHOREmap, which implements a likelihood ratio test statistics that converts mapping-by-sequencing data into confidence-mapping intervals [7]. These mapping intervals represent the region, in which causal candidates reside at a given confidence level *P *(here *P=*0.99). As we assume that mutations are randomly introduced into the genome, the number of CAMs is linearly correlated with the length of mapping intervals, which we used to quantify the outcome of a mapping-by-sequencing experiment. Though marker density positively impacts on mapping resolution, inclusion of markers that cannot be accessed with the actual sequencing methods or that have been falsely included can have severe local effects on the precise determination of mapping intervals [7]. The marker set we used consisted of 291,973 markers, after discarding closely linked polymorphisms and those in repetitive regions from the complete set of differences between *Arabidopsis *accessions Columbia (Col-0) and Landsberg *erecta *(L*er*) [21] (Material and methods).

#### Interplay of pool size and genome-wide coverage

**Figure 2:**
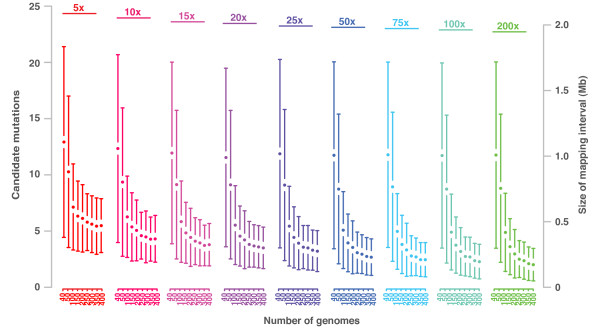
**Results of mapping-by-sequencing with outcross populations**. Pools of 40 to 400 individuals (colored blocks) were sequenced with increasing coverage ranging from 5x to 200x. For each combination of pool size and coverage we simulated 500 independent populations and performed a mapping-by-sequencing analysis on each of them. Average mapping interval size and its standard deviation as well as the imputed number of candidate mutations within mapping region are shown on right and left y-axis, respectively. The initial number of mutations per genome was 1,400.

Outcross populations were simulated with 40 to 400 mutant genomes. Next generation sequencing was simulated at various genome-wide coverage levels ranging from 5x to 200x. Each combination of pool size and coverage was independently repeated for 500 times. For each dataset we performed a SHOREmap analysis and assessed the size of the final mapping intervals (Figure 2). Overall, the sizes of the mapping intervals were remarkably variable. This variation was lower for pools with more recombinants as compared to pools with fewer recombinants. As expected, the number of recombinants also strongly influenced mapping resolution. For example, at an average genome-wide coverage level of 15x, pools with 200 recombinants yielded an average interval size of 381 (± 222) kb, whereas pools with 50 recombinants generated interval sizes of 783 (± 567) kb on average. Like in conventional mapping experiments, the decrease in the size of the mapping interval was not linear. The first indication of saturation was observed at a sequencing coverage of 5x to 15x, where increasing the pool size beyond 350 recombinants did not improve the interval size.

In contrast to pool size, coverage alone had only a small effect on size and variation of mapping intervals. Pools of 100 recombinants, which were sequenced at 15x, yielded an average interval size of around 500 (± 310) kb, as compared to 419 (± 298) kb at a coverage of 200x. The reason for the weak impact of coverage on the size of the mapping interval is the large number of markers, which are distributed throughout the genome and allow for an accurate assessment of allele frequencies even at low coverage levels.

Assuming 1,400 mutagen-induced mutations per genome, the average number of CAMs was around five for pools of >100 recombinants sequenced at an average genome-wide coverage of 25x. In practical application, additional prioritization by functional annotation and location of mutations in the interval has the potential to reduce this low number of CAMs to one outstanding candidate only [1].

### Mapping-by-sequencing with backcross populations

Conventional genetic mapping requires a cross of the mutant to a diverged genome. In addition to genetic variation, this introduces phenotypic variation, which can interfere with the recognition of subtle phenotypes. Moreover, if the mutagenesis was performed in a complex (transgenic or otherwise mutagenized background) this background needs to be introgressed into the diverged genome, if tedious genotyping for the presence of first site mutations within all recombinants is to be avoided.

In order to bypass these obstacles, it has been suggested to use F_2 _populations derived from backcrossing the mutant plant to the non-mutagenized progenitor as mapping populations [5,6,13]. Within backcross populations all mutagen-induced mutations segregate, except for the causal and closely linked mutations, which are fixed in the mutant pool by selecting for the mutant phenotype. Thus, selection for fixed differences between the mutant pool and its genetic background reduces the number of putative causal changes considerably. To quantify results of each simulation, we used the number of homozygous differences between the mutant pool and the background. However, the absolute number of homozygous mutations greatly depends on the definition and settings of parameters used for their identification. As sequencing errors can introduce wild-type alleles at otherwise homozygous loci, selecting only those positions without reads that support the wild-type allele excludes some of the real homozygous mutations. On the other hand, including positions with support for wild-type alleles will introduce false positives. In order to allow comparisons across samples, we defined and applied thresholds, which are adjusted to pool size and sequencing coverage (Materials and methods). Backcrossing was simulated by crossing a single mutant plant to its isogenic parent followed by one generation of inbreeding to establish a BC_1_F_2 _mapping population (see (I) in Figure 1).

#### The interplay of pool size and genome-wide coverage in BC_1_F_2 _populations

**Figure 3:**
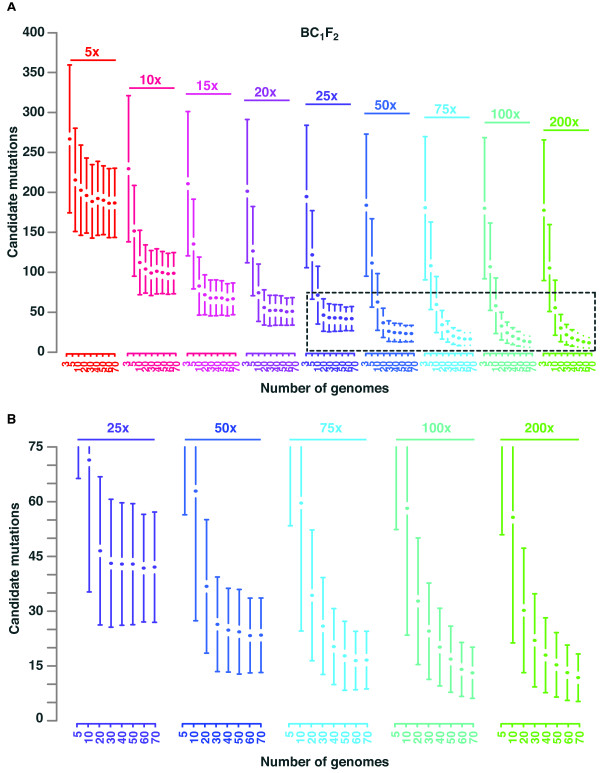
**Results of mapping-by-sequencing with backcross populations**. (**A**) Pools of 3 to 70 BC_1_F_2 _individuals (colored blocks) were sequenced with increasing coverage ranging from 5x to 200x. For each combination of pool size and coverage we simulated 500 independent populations and performed a mapping-by-sequencing analysis on each of them. Average number of candidate mutations with one standard deviation is shown on the y-axis. The initial number of mutations per genome was 1,400. (**B**) Zoom in on the framed region in panel A. Pools with three recombinants are not shown.

We simulated BC_1_F_2 _populations with 3 to 70 mutants for high and low mutation rates separately. Sequencing was simulated at different coverage levels, ranging from 5x to 200x. For each combination of pool size and coverage level, we simulated 500 independent mapping populations and scored the number of homozygous mutations (Figure 3). Mutations that are not fixed, but are close to fixation have a high probability to appear as fixed in the sequencing data. This effect becomes stronger at low coverage levels, where the reduced number of reads does not allow identifying low frequencies of wild-type alleles. As expected, more recombinants reduced the average number of homozygous candidate mutations. Sequencing pools with 30 recombinants at coverage of 25x revealed 43 (± 18) CAMs on average. Like for outcross populations, the variation of CAMs was high in pools with few recombinants, but got reduced in larger pools.

In great contrast to outcross populations, we observed immediate saturation of the number of CAMs while increasing pool size. For example, pools with 20 mutants sequenced at a coverage level of 20x revealed 56 (± 22) CAMS on average. Pools with 70 mutants, which were sequenced with the same sequencing effort, revealed almost the same number. In general, for coverage levels of <25x, we observed no reduction in the number of CAMs when increasing the pools beyond 20 recombinants. This suggests that low-fold sequencing lacks the power to make use of the compliment of recombination in the pool and more sequencing is required to exploit all recombination events. In agreement, we still observed a decrease in CAMs for deeply sequenced samples (200x) when increasing pool size from 60 to 70. This illustrates the mutual importance of both pool size and coverage. This trend was also observed when introducing a low mutation load (Figure S1 in Additional file 1).

#### Effects of successive backcrossing

**Figure 4:**
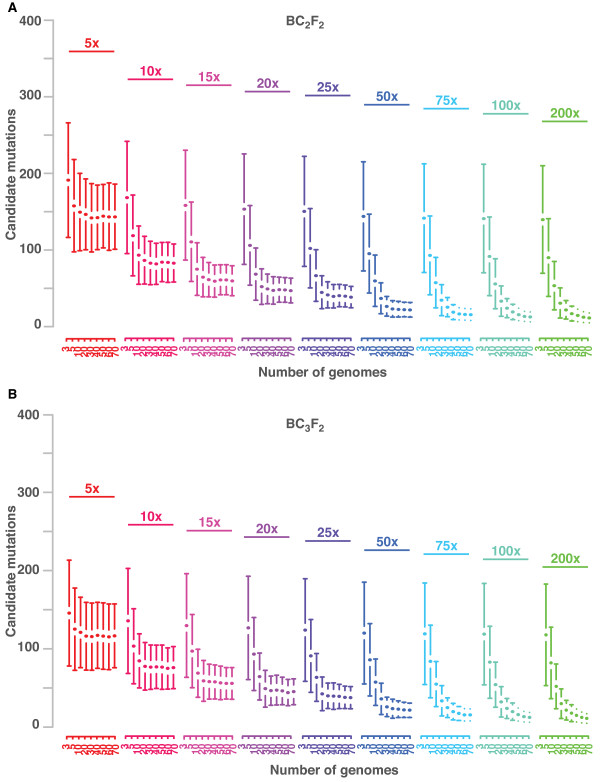
**Effect of coverage and pool size on BC_2_F_2 _and BC_3_F_2 _backcross populations**. (**A**) Pools of 3 to 70 BC_2_F_2 _individuals (colored blocks) were sequenced with increasing coverage ranging from 5x to 200x. For each combination of pool size and coverage we simulated 500 independent populations and performed a mapping-by-sequencing analysis on each of them. Average number of candidate mutations with one standard deviation is shown on the y-axis. The initial number of mutations per genome was 1,400. (**B**) Outcome of the same analysis with BC_3_F_2 _recombinants.

In a series of simulations, we increased the number of backcross generations up to three before establishing a mapping population (see (II) in Figure 1). In total, mapping-by-sequencing of 81,000 BC_2_F_2 _and BC_3_F_2 _populations were compared to the prior analysis of BC_1_F_2 _pools. As expected, additional backcrosses reduced the variation of CAMs in pools with a few plants (Figure 4). In particular, when genome-wide coverage or the number of mutants was limited, additional rounds of backcrossing helped to reduce the number of CAMs. However, pools with a reasonable number of recombinants sequenced with sufficient coverage did not improve with additional backcrosses.

Backcross populations are usually derived from one single mutant plant and its isogenic parent. However, generation of a backcross population based on multiple mutant siblings, all of which are crossed to their isogenic parent, introduces additional variation around the causal locus. Here, we simulated the generation of backcross populations with three mutant siblings and compared the mapping outcome to our previous results, which were based on one mutant parent only (Figure S2 in Additional file 1). The improvement in mapping resolution was very limited and restricted to pools with few mutants only.

#### Effects of mis-scored plants

Phenotyping complex or subtle phenotypes can lead to mis-scored plants. Such plants introduce wild-type alleles at the causal candidate locus and severely interfere with genetic mapping. In order to study the effect of mis-scored recombinants, we simulated different rates of mis-scored plants ranging from 1% to 6% within a population of 50 BC_1_F_2 _mutants sequenced at 50x (Materials and methods). Compared to previous results, pools with 1% to 2% false scored plants yielded 82% and 145% more CAMs, respectively (Figure S3 in Additional file 1). This illustrates that even small errors in the phenotype can have severe effect on mapping-by-sequencing based on backcross populations.

### Direct sequencing of mutant genomes

As an alternative to bulk segregant analysis, individual mutant genomes can be sequenced directly (see (III) in Figure 1). However, the large number of background mutations interferes with the unambiguous identification of causal mutations. Backcrossing removes some of these background mutations [14,15]. Here, we analyzed mutant genomes after one to three rounds of backcrossing. Mutants that are selected from backcross populations will generally yield fewer CAMs. The theoretical fraction of the recurrent parental genome after *n *rounds of backcrossing is $\frac{2^{n+1}-1}{2^{n+1}}$[22]. Our simulated populations closely followed the expected percentage and showed an average reduction of foreground genome by 12.8% and 6.8% in BC_2 _and BC_3_, respectively. As expected, direct sequencing yielded more CAMs than in our bulk segregant analyses. For example, across all coverage levels, pools with no more than three BC_1_F_2 _mutant individuals showed less than half of the CAMs as compared to direct sequencing of BC_1_F_2 _individuals, illustrating the power of bulk segregant analysis.

### Paired-end *versus *single-end sequencing

Many of the new sequencing technologies allow sequencing of one or both ends of DNA clones. Paired-end sequencing enables access to (the borders of) repetitive sequences, which increases the number of markers and mutations that can be analyzed. Even though single-end sequence reads might not be able to explore the same genomic space as paired-end sequence reads, they are independent of each other. In bulk segregant sequencing, independent reads are counted to estimate allele frequencies (see (IV) in Figure 1). If both reads of a pair align to different markers, they cannot contribute twice to the estimation of allele frequencies as they carry the same genetic background (ignoring the very rare cases, where read pairs span recombination events). If two single-end reads overlap with markers, both contribute to the estimation of allele frequencies as they have been sampled independently. It is thus not obvious whether paired-end or single-end sequencing is advantageous for mapping-by-sequencing.

**Figure 5:**
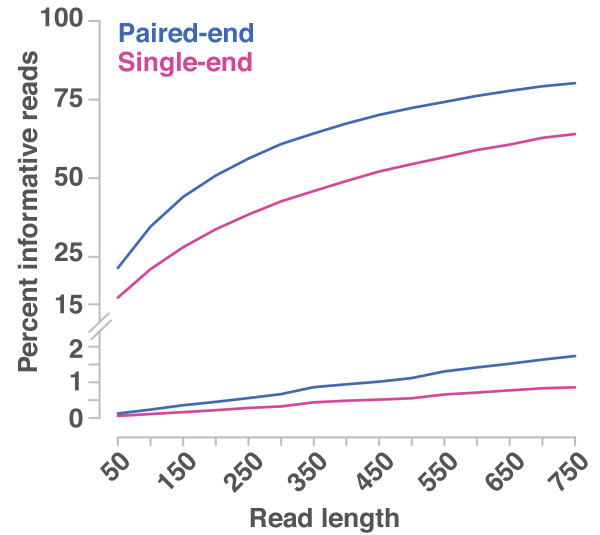
**Percentage of informative reads for different sequencing read lengths and types**. Reads or read pairs, respectively, can only contribute to mapping-by-sequencing if their alignments overlap with at least one marker or mutations (informative reads). The number of informative reads from single-end and paired-end sequencing are shown in purple and blue, respectively. The lower graphs refer to a mutation density, which is typical for backcross populations (here, 1,400 mutations per mutant genome). The upper graphs refer to the number of marker in outcross populations (281,668 and 291,973 for single-end and paired-end sequencing).

We have compared the efficiency of single and paired-end reads by counting the number of randomly generated read or read pair alignments that overlap with predefined marker and CAMs sets. A read, respectively a pair, was scored as informative if it was uniquely aligned to at least one or more markers. The lengths of the simulated reads ranged from 50 bp to 750 bp to cover a wide range of next generation sequencing read lengths (Figure 5). Reads, which align equally well to multiple regions in the genome, are excluded for further analysis. Increased read length span some of the short repeats and thus allows aligning more reads uniquely [23,24].

For the analysis of mapping-by-sequencing with outcross populations, we defined two sets with 291,973 and 281,668 markers for single-end and paired-end sequencing, respectively, in order to take the different mapping properties into account. Depending on the read length, paired-end sequencing featured between 25% and 78% more informative read pairs. Consequently, it would require between 25% and 78% more single-end reads in order to end up with the same number of informative reads. This calculation allows for a cost comparison of mapping-by-sequencing for single and paired-end sequencing based on actual sequencing costs. However, as paired-end sequencing enables the analysis of parts of the otherwise inaccessible DNA, it might be advantageous to sequence both ends, even if this would be more expensive. In particular if combined with mutation identification, paired-end sequencing has a higher chance not to miss the causal mutation. We repeated this exercise for mapping-by-sequencing based on backcross populations that were simulated with 1,400 mutations in the genome. Here, paired-end sequencing featured between 95% and 119% more informative read pairs.

### Application of mapping-by-sequencing simulations to model crop species

Mapping-by-sequencing has already been successfully applied to crop species, like rice and polyploid wheat [5,16,25]. As the size of some of the crop genomes can be as large as multiple Gb, an informed decision on the experimental design of mapping-by-sequencing seams even more important for such species. Here, we explored the power of Pop-seq simulator to address questions about the experimental design of mapping-by-sequencing experiments in rice and barley, where mapping-by-sequencing starts to become part of standard molecular toolbox.

#### Mapping-by-sequencing in the crop model species rice

First, we estimated the recombination frequency and landscape of rice by combining two publically available rice RIL populations [26,27]. Further, we selected a publically available set of 139,244 marker for the simulation of outcross populations markers [28]. Similarly to *Arabidopsis*, we randomly introduced 2,222 mutations (1 every 171 kb), of which one was selected to be causal. Based on this, we simulated mapping-by-sequencing using both outcross and BC_1_F_2 _backcross populations with 50 to 400 and 10 to 80 mutant genomes, respectively. Sequencing of these pooled genomes was simulated at various genome-wide coverage levels ranging from 10x to 100x. Each combination of pool size and coverage was simulated for 300 times.

Overall, we observed very similar trends for mapping-by-sequencing in rice as compared to *Arabidopsis *(Figure S4 and S5). Changes in the genome-wide coverage affected the outcome of backcross populations more than outcross populations and pools with very low number of recombinants drastically suffered from the lack of recombination. Outcross populations with 150 mutant recombinants sequenced with not more than 20x featured <3 CAMs on average in our simulations. In contrast, backcross populations consisting of 50 mutants, which were sequenced at a genome-wide coverage of 50 yielded around 10 CAMs on average.

In general, the greater genome size of rice as compared to *Arabidopsis*, was counteracted by an enriched recombination frequency allowing for similar conclusions on the experimental design in rice as in *Arabidopsis*.

#### Mapping-by-sequencing based on targeted enrichment sequencing

As-of-today, the large genome sizes of crop species like the one of *Hordeum vulgare *(barley) make whole-genome resequencing as part of mapping-by-sequencing an expensive and risky approach. To address this general problem genome-complexity reduction methods, like transcriptome sequencing, restriction site associated DNA sequencing, or targeted enrichment sequencing, have been proposed [29-32]. For example, targeted enrichment sequencing has been proven to be suitable for mapping-by-sequencing already [7].

Here, we simulated targeted enrichment sequencing of approximately 60 Mb of the barley genome. This includes the simulation of deep sequencing at selected regions of the genome, but at the same time the simulation excludes the rest of the genome from sequencing. Even though enrichment sequencing has a high chance to exclude the causal mutation from the actual sequencing data, mapping-by-sequencing based on enrichment sequencing will guide subsequent fine-mapping efforts.

The design of the enrichment reduced the set of genome-wide marker as defined between the two cultivars Morex and Barke from 11,371,643 to 164,492 markers, which are accessible through our enrichment sequencing [33]. Mapping populations were simulated with 50 to 600 mutant plants selected from F_2 _outcross populations and were based on the recombination frequency and landscape for barley as observed in the Oregon Wolfe Barley mapping population [34]. Sequencing was simulated at coverage levels of 100x to 1,000x reflecting the high coverage gained in enriched regions. Each combination of pool size and coverage was simulated for 300 times.

Overall, the reduced recombination frequency in barley as compared to the other species resulted in large mapping intervals (Figure S6). Similarly to the observations for the other two species, increased coverage had only a minor effect on the results of outcross population-based mapping-by-sequencing, but an increase in the number of mutants can have a strong effect on the size of the mapping interval. Simulation of mapping populations with 400 mutants that were sequenced with an average coverage 200x at the enriched regions resulted in mapping intervals with an average size of 3.2 Mb.

## Conclusions

Identification of a wide range of mutagen-induced phenotypes founded the basis of genetic research in *Arabidopsis *in the last century [35]. Next generation sequencing now revived and accelerated this successful concept of forward genetics as it allows for instant identification of CAMs. This speed-up in genetic mapping now opens new avenues even for more complex phenotypes. We have addressed several questions about the design of mapping-by-sequencing in a large simulation study and summarize our suggestions for reasonable and successful experiments (Table 1).

**Table 1:** Suggestions for the design of mapping-by-sequencing experiments

	Outcross populations	Backcross populations	Direct sequencing	Deep candidate resequencing (dCARE)
Generation	F_2_	BC_1_F_2_	BC_1-3_F_2_^a^	n/a
Mutants (n)	Approximately 150	Approximately 50	1	As many as possible
Optimal coverage	>25	Approximately 50	>25	n/a
Sequencing type	Paired-end	Paired-end	Paired-end	Single-end

^a^Depending on mutation rate.

*In-silico *simulations have the tendency to be too optimistic in their predictions, as simulations only consider known variables, but additional noise, which is common to real data, might not be simulated appropriately. Differences between real mapping-by-sequencing experiments and our simulations include differences in the number of recombination and mutations. As recombination events can be influenced by changes in the environment as well as the genetic background, our empirical data, which was based on limited environments and genetic backgrounds, will not always fit the real recombination landscape [36]. However, variation in recombination is subtle, and our results are in agreement with published mapping-by-sequencing experiments [1,3].

We have analyzed the effect of genome-wide coverage and recombinants on the performance of mapping-by-sequencing. Our results revealed that coverage and recombinants are interconnected and rely on the crossing scheme. Mapping-by-sequencing with backcross populations requires higher coverage for optimal mapping results as compared to outcross populations. This difference comes from the drastic difference in the number of markers (or mutations) between these two types of populations. Outcross populations contain hundreds of thousands of natural markers, which are much denser than the expected recombination frequency. Thus, sliding-window-like approaches can combine the information from neighboring markers, and establish precise allele frequency estimates. Even though the average coverage for sliding-window-based methods can be low, subsequent identification of mutations limits the minimal coverage required of such experiments. In order to avoid the risk of missing the causal mutation, it is not recommended to target coverage levels of <25x for mapping-by-sequencing experiments.

As a consequence of the reduced analysis power, mapping-by-sequencing with backcross populations cannot make use of the full complement of recombination. In contrast to outcross populations, coverage has a large impact on the final number of CAMs.

In order to prioritize the final set of CAMs, their putative effects on genes can be examined. An attractive, additional way to further filter the final set of CAMs is generating more sequencing data. Hartwig *et al*. suggested deep sequencing of PCR amplicons (or deep candidate resequencing, dCARE) to locally increase the read coverage up to multiple thousands of reads [6]. This has the advantage of avoiding expensive whole-genome sequencing, while providing an exact count on allele frequencies at each CAM. If dCARE is considered, pooling many recombinants, even more than the whole-genome sequencing can resolve, is recommended.

Irrespective of the demands in coverage and number of mutants, decisions on the experimental setup should consider the phenotype. Complex phenotypes, which can be affected by the presence of natural variation will benefit from isogenic mapping strategies, as mis-scored phenotypes can have severe effects on the mapping result.

As an alternative to bulk segregant analysis, we also analyzed direct sequencing of individual genomes of backcross populations. Each successive backcross reduces the foreground genome and the number of CAMs. However, it requires multiple backcross generations before the number of CAMs is as low as in bulk segregant analyses and still tedious rough mapping may be required [15]. Our study suggests that multiple rounds of backcrosses can be avoided by pooling multiple genomes.

The genome-wide mutation rate of radiation mutants is reported to be significantly lower as compared to chemically induced mutants [37]. Direct sequencing of mutants with fewer, but putatively more severe mutations can simplify the interpretation of whole-genome analysis of directly sequenced mutant genomes.

Another way to reduce the number of CAMs is sequencing multiple independent alleles of the same mutant, which was shown recently for the first time [16]. This strategy does not reduce the number of CAMs in individual genomes, but most of the CAMs can be ignored as they affect genes only in one of the alleles. The chance of finding unrelated genes with mutations in different alleles is very low [16].

We have extended our analysis on the design of mapping-by-sequencing experiments to rice and barley, which involved the simulation of targeted enrichment sequencing. While the larger genome of rice as compared with *Arabidopsis *was counteracted by an elevated recombination frequency, the interval sizes for barley were drastically increased, which is in agreement with its large genome size.

The outcome of the simulations relies in large parts on the recombination frequency and hotspots and different varieties might feature different recombination landscapes. Thus, in order to study mapping-by-sequencing in different species or other crossing schemes, we propose to repeat the simulations with the appropriate recombination frequency and landscape. Our Pop-seq simulator is available for download and will ease such an application (see Materials and methods).

## Materials and methods

### Simulation of recombined individuals and mapping populations

Simulation of *in-silico *mutant genomes started by creating an initial mutant genome with 700 or 1,400 randomly placed, homozygous mutations, respectively. One of those was randomly selected as causal mutation. Wild-type genomes were simulated without any mutation. In order to simulate offspring genomes, we combined recombined haploid genomes from one or two virtual parents. Offspring genomes were used as parents for further crosses. The actual number of recombination per meiosis for each chromosome was randomized based on the distribution of recombination events in *Arabidopsis*, these empirical determined recombination frequencies were derived from a cross between *Arabidopsis *Col-0 and Fei-0 [17]. It was calculated by

$$X\;\sim \;Trinomial\left(n,\;\left[p_{1},p_{2},p_{3}\right]\right),$$

where *p_1_*, *p_2 _*and *p_3 _*are the observed frequencies of none, one, and two or more recombination per chromosome per meiosis. The location of each recombination was selected after the observed frequencies over each marker along the chromosome and placed in between two adjacent markers. The probability of a recombination at position *x_ij _*between two adjacent markers was calculated by

$$\left\{2,3,\;..,\text{k}\right\}p_{\left(x_{j_{i}}\right)}=\frac{p\left(m_{i}\right)}{l_{i}},\;i=\;,\;j_{i}=\left\{1,2,..,l_{i}\right\},$$

where *i*, *j*, *k*, and *l *are the marker, base pair, total number of markers, and length between adjacent markers, respectively.$p_{\left(m_{i}\right)}$ is the observed probability of recombination in between marker *m_i _*and *m_i-1_*. The location of additional recombination events was modeled after a gamma distribution in order to take crossing over interference into account. Both gamma distribution parameters scale and shape were chosen such that the resulting distribution followed the empirical data. Plants with homozygous causal mutations were labeled as mutant phenotypes.

### Simulation of whole-genome sequencing

Accurate simulation of whole-genome sequencing of bulks and individual genomes needs to consider the total number of alignments per marker, the parental allele frequencies, and sequencing errors. To incorporate the variation in the number of alignments per marker, we assigned a prior normalization *n *value to each marker position based on the observed coverage in real resequencing experiments of *Arabidopsis *wild-type [21]. The value describes the ratio of observed coverage at single marker in relation to the genome-wide average. Actual number of reads at each marker position *c_i _*per sequencing simulation was then calculated by

$$c_{i}\;\sim \;Multinomial\left(m,\;\left[n_{1},n_{2},..n_{k}\right]\right),$$

where *m*, *n*, and *k *are the total number of reads, normalized coverage probability per marker, and the total number of markers, respectively. Then, we used the allele frequency *a_1_*, and *a_2 _*within the population under investigation and assigned each read *r_i _*to one of the parental alleles by

$$r_{i}\;\sim \;Trinomial\left(c_{i},\;\left[a_{1},a_{2},s\right]\right),$$

where *a_1_*, *a_2 _*and *s *are the allele frequency of mutant, allele frequency of wild-type and sequencing error. We used a constant sequencing error rate of 0.3% [7]. The frequency of different types of sequencing errors in Illumina sequencing is non-randomly distributed [19], however as this would have a limited impact on our simulations, we did not address this fact here.

### Selection of homozygous mutations

Predictions of homozygous mutations are affected by pool size and coverage. In order to define a uniform threshold for the detection of homozygous mutations across all deeply and shallowly sequenced pools with a few or many plants, we introduced two thresholds respecting pool size and sequencing coverage. First, we calculated the mutant allele frequency at loci where one single wild-type chromosome is present, defined as

$$g_{f}=1-\left(\frac{\left[m*2\right]+1}{n*2}\right),$$

where *m *and *n *are the number of mis-scored and total mutants in the pool, respectively. For the second threshold, we calculated the mutant allele frequency as estimated by the short read alignments, where one alignment is sampled from a non-mutant chromosome, defined as

$$r_{f}=1-\left(\frac{\left\lceil c_{p}*e\right\rceil +1}{c_{p}}\right),$$

where *c_p _*is the actual coverage at position *p *and *e *is the estimated sequencing error frequency of 0.3% [7]. Only mutations with mutant allele frequencies greater than *g_f _*and *r_f _*have been considered as homozygous mutations.

### High quality marker selection

For all simulations based on outcross populations we defined a high quality marker set based on resequencing data of *Arabidopsis *L*er *[21]. All SNPs with a resequencing quality score <25 were discarded, as well as SNPs that overlap with regions with different copy number between the parents as predicted by the resequencing. Further we iteratively removed SNPs, which were closer than 50 bp. This yielded 291,973 high quality markers.

### Comparison of single-end and paired-end sequencing

Single and paired-end sequencing was simulated with reads ranging from 50 bp to 750 bp in length. Insert length for paired-end sequencing was simulated with three times the read length. For each combination of read length and sequencing type 100,000 random alignments locations were chosen. The read length defined the end of the alignment. The actual location of the read pair was defined by read length and insert site. If the simulated alignments overlapped with one or more markers the alignment was scored as informative.

### Implementation of simulation pipeline

Our pipeline for simulating recombinant genomes and their sequencing, Pop-seq simulator, is available at sourceforge [38]. Recombination frequency and landscape are specified by configuration files, which we provide for all simulations performed in this study.

## List of abbreviations

Bp: base pair; CAMs: candidate mutations; dCARE: deep candidate resequencing; FNR: fast-neutron radiated; Gb: giga bases; kb: kilo bases; Mb: mega bases.

## Competing interests

The authors declare that they have no competing interests.

## Authors' contributions

KS and GVJ designed the study. GVJ, VP, KJVN, and KS implemented the simulation pipeline. GVJ, JK, and KS performed data analysis. PS and DW provided biological resources. KS and GVJ wrote the manuscript. All authors read and approved the final manuscript.

## Authors' information

KS and DW published the first mapping-by-sequencing analysis and software for next generation sequencing-based bulk segregant analyses. Their labs perform mapping-by-sequencing analyses on a regular basis.

## Additional data files

The following additional data are available with the online version of this article. Additional file 1 describes supplementary figures.
